# Autophagic Impairment Contributes to Systemic Inflammation-Induced Dopaminergic Neuron Loss in the Midbrain

**DOI:** 10.1371/journal.pone.0070472

**Published:** 2013-08-06

**Authors:** Hui-Fen Zheng, Ya-Ping Yang, Li-Fang Hu, Mei-Xia Wang, Fen Wang, Li-Dan Cao, Da Li, Cheng-Jie Mao, Kang-Ping Xiong, Jian-Da Wang, Chun-Feng Liu

**Affiliations:** 1 Department of Neurology, Second Affiliated Hospital of Soochow University, Suzhou, Jiangsu, China; 2 Institute of Neuroscience, Soochow University, Suzhou, Jiangsu, China; 3 Department of Neurology, Yixing People’s Hospital, Yixing, Jiangsu, China; Emory University, United States of America

## Abstract

**Background:**

Neuroinflammation plays an important role in the pathogenesis of Parkinson’s disease (PD), inducing and accelerating dopaminergic (DA) neuron loss. Autophagy, a critical mechanism for clearing misfolded or aggregated proteins such as α-synuclein (α-SYN), may affect DA neuron survival in the midbrain. However, whether autophagy contributes to neuroinflammation-induced toxicity in DA neurons remains unknown.

**Results:**

Intraperitoneal injection of lipopolysaccharide (LPS, 5 mg/kg) into young (3-month-old) and aged (16-month-old) male C57BL/6J mice was observed to cause persistent neuroinflammation that was associated with a delayed and progressive loss of DA neurons and accumulation of α-SYN in the midbrain. The autophagic substrate-p62 (SQSTM1) persistently increased, whereas LC3-II and HDAC6 exhibited early increases followed by a decline. *In vitro* studies further demonstrated that TNF-α induced cell death in PC12 cells. Moreover, a sublethal dose of TNF-α (50 ng/ml) increased the expression of LC3-II, p62, and α-SYN, implying that TNF-α triggered autophagic impairment in cells.

**Conclusion:**

Neuroinflammation may cause autophagic impairment, which could in turn result in DA neuron degeneration in midbrain.

## Introduction

Multiple lines of evidence have implicated neuroinflammation in the onset and progression of Parkinson’s disease (PD) and other neurodegenerative disorders [1]. Post-mortem analyses of PD patients and animal models have revealed activated microglia and accumulation of inflammatory mediators in substantia nigra (SN) [2]–[4]. Anti-inflammatory drugs have been reported to reduce dopaminergic (DA) neuron loss in various PD models [5], [6]. Inflammatory cytokine gene polymorphisms are associated with increased risk of PD [7]–[9]. Moreover, the SN is densely populated with microglia and is susceptible to inflammatory insults [10]. Systemic inflammation has been reported to trigger microglial activation and to contribute to chronic degeneration in PD [11]. LPS is a major component of the outer membrane of Gram-negative bacteria, and it produces neurotoxicity in the presence of microglia [12], [13]. Researchers have demonstrated that a single intraperitoneal injection of lipopolysaccharide (LPS, 5 mg/kg) into C57BL/6J adult mice caused a delay of neuronal loss in SN. This unique property mimics the chronic progression of PD. This is helpful in studying inflammation-related neurodegeneration and its related cellular and molecular mechanisms [14].

The formation of Lewy bodies is another pathological hallmark of PD [15]. α-synuclein (α-SYN) is the main component of Lewy body and is degraded via the ubiquitin-proteasome system (UPS) and the autophagy-lysosomal system (ALP) [16]. In mammals, three types of autophagy have been described: macroautophagy, microautophagy, and chaperone-mediated autophagy. All uses of the term “autophagy” in this study refer to macrophagy unless otherwise noted. When the UPS is not effective, the ALP will be activated in a histone deacetylase 6 (HDAC6)-dependent manner under certain conditions [17]. Autophagy impairments worsen the clearance of misfolded proteins, contributing to the development of neurodegenerative disease [18], [19]. In recent years, mounting evidence has linked dysfunctional autophagy to PD pathogenesis. However, the underlying mechanisms of this impairment in autophagy must be elucidated.

Given that intraperitoneal LPS injection caused DA neuron losses in SN and that autophagic deficits might be involved in DA neuron degeneration in PD, we hypothesized that alterations in autophagic function may occur during inflammatory processes. These changes in autophagy may subsequently result in endogenous α-SYN accumulation and neuronal loss in the midbrain. Thus, the present study was designed to examine the possible changes in autophagic activity during neuroinflammation caused by LPS injection in mice. In addition, as PD incidence increases with age, whereas autophagic activity decreases, both young (3-month-old) and aged (16-month-old) mice were used to reveal the age-related changes in DA neuron loss and autophagic function.

## Materials and Methods

### Animals and Experimental Procedure

Male C57BL/6J mice were purchased from Shanghai Laboratory Animal Center (China). Animal care and procedures were performed in accordance with the Laboratory Animal Care Guidelines approved by Shanghai Institutes for Biological Sciences of Chinese Academy of Sciences. Permit numbers: SCXK (HU) 2007–0005; SYXK (HU) 2008–0049. The experimental protocols were approved by the Institutional Animal Care and Use Committee of Soochow University. Considering the age-dependent changes of inflammation and autophagy, both 3- and 16-month-old mice were used in this study to represent young and aged conditions. Mice were housed under a 12 h light/dark cycle with free access to food and tap water. A single intraperitoneal injection of 5 mg/kg LPS (Sigma, St. Louis, MO, USA) was used to establish a parkinsonian model according to a previous report [11]. Animals were randomly divided into four groups (n  = 8–10 each group): (I) 16-month-old mice treated with saline (16-C); (II) 16-month-old mice treated with LPS (16-L); (III) 3-month-old mice treated with saline (3-C); and (IV) 3-month-old mice treated with LPS (3-L).

### Immunohistochemistry

Mice were euthanized and transcardially perfused with 4% paraformaldehyde in 0.1 M phosphate buffered saline (PBS). Brains were harvested, and midbrain sections containing SN (Bregma −2.8 to −3.8 mm) were then extracted. The brains were embedded in paraffin and cut into 5 µm sections. Endogenous peroxidase activity was inactivated with 0.3% H_2_O_2_ for 30 min. After washing with PBS, sections were incubated in a 2% goat serum/0.1% Triton X-100 in 0.1 M PBS for 1 h, followed by incubation with primary antibodies against proteins of interest: mouse monoclonal anti-tyrosine hydroxylase (TH) (1∶1000 Sigma, USA), anti-ionized calcium binding adaptor molecule 1 (Iba1, 1∶100, Abcam, Hong Kong), rabbit anti-LC3 (1∶50 Abcam, Hong Kong), and rabbit anti-p62 (1∶50 ZNEO, Japan) at 4°C overnight. Then, slides were incubated with the corresponding Alexa Fluor- or HRP-conjugated secondary antibodies for 1 h at room temperature. For HRP-conjugated antibodies, immunoreactivities were developed with diaminobenzidine solution. HRP sections were observed and photographed using a Zeiss microscope (AXIO SCOPE A1, Zeiss Corp, Germany); Alexa Fluor sections were observed using a confocal microscope (LSM700, Zeiss Corp). DA neuron loss was assessed by counting the number of TH-IR neurons in every sixth section by two researchers blind to the treatment. Data are expressed as a percentage compared to the saline-injected controls.

### Western Blot Analysis

Tissues and cells were homogenized in ice-cold lysis buffer (50 mM HEPES, 5 mM EDTA, 150 mM NaCl, 0.5 mM ATP, and 0.1% TritonX-100, pH 7.5) with protease inhibitor cocktail tablets (Roche, Germany). The lysates were centrifuged at 14,000 g for 20 min at 4°C. The resulting supernatants were collected, and protein concentrations were determined using the BCA protein assay kit (Pierce, Rockford, IL, USA). Proteins were separated on 10–12% sodium dodecyl sulfate-polyacrylamide gels where applicable and transferred onto nitrocellulose membranes. Blots were blocked with 5% nonfat dry milk in PBST (3.2 mM Na_2_HPO_4_, 0.5 mM KH_2_ PO_4_, 0.1% Tween-20, and pH 7.4) for 1 h and then incubated with primary antibodies, such as rabbit anti-α-SYN (1∶1000 Cell Signaling, Boston, MA, USA), rabbit anti-LC3B (1∶1000 Abcam, Hong Kong), rabbit anti-p62 (1∶1,000 ZNEO, Japan), rabbit anti-HDAC6 (1∶500 Bioworld Technology, Louis Park, USA), mouse β-actin, and mouse GAPDH (Sigma, USA) at 4°C overnight. Then, blots were washed with PBST buffer and incubated with HRP-conjugated secondary antibodies for 2 h. Blots were then visualized using ECL chemiluminescence (Thermo Company, West Chester, PA, USA). Band densities were analyzed with Image Quant (Bio-Rad, USA).

### Electron Microscopy

The midbrains were post-fixed, and then, SN regions were punched out and processed using standard EM osmication with en bloc staining procedures. Briefly, blocks were flat embedded in epon, attached to beam capsules, trimmed, and cut into ultrathin sections using an ultramicrotome. Then, sections were stained with uranyl acetate and lead citrate. Finally, sections were scanned and observed with a JEM 1010 transmission electron microscope.

### Tissue TNF-α Measurement

To determine the tumor necrosis factor alpha (TNF-α) level in ventral midbrain, tissues were homogenized in lysis reagent (Sigma, USA) on ice, and the resulting supernatants were collected for Enzyme-Linked Immunosorbent Assay (ELISA) (R&D Systems, USA) according to the manufacturer’s instructions. Absorbance was measured at 450 nm using a microplate reader (Tecan M200, Grodig, Austria).

### Cell Culture and Viability Determination

PC12 cells (ATCC) were grown in Dulbecco’s modified Eagle’s medium (DMEM) supplemented with 10% fetal bovine serum and 1% penicillin/streptomycin in a 5% CO_2_ atmosphere at 37°C. Cells were plated onto 96-well plates for cell viability determination or 35 mm dishes for other assays. Cells were exposed to TNF-α (R&D Systems, USA) for 24 h. Cell viability was determined with an MTT (3-[4, 5-dimethylthiazol-2-yl]-2, 5-diphenyltetrazolium bromide) reduction assay. MTT solution (0.5 mg/ml, Sigma, USA) was added into each well at the end of treatment, and plates were incubated at 37°C for 4 h. The absorbance was finally measured at 570 nm with a reference wavelength at 630 nm, using a microplate reader (Tecan M200, Grodig).

### Reverse Transcription Polymerase Chain Reaction (PCR)

Total RNA was extracted using TRIzol® reagent (Invitrogen, Carlsbad, CA, USA). Equal amounts of RNA (1 µg) were reversely transcribed into cDNA using Revert Aid First Strand cDNA synthesis kit (Fermentas). An equal volume of cDNA product was amplified using PCR Master Mix kit (Fermentas), with the following primers (Genscript, Nanjing, China): p62, 5′-CAG GCG CAC TAC CGC GAT GA -3′(forward), 5′-TCG CAC ACG CTG CAC AGG TC-3′(reverse); GAPDH (rat), 5′-CAA GGT CAT CCA TGA CAA CTT TG-3′(forward), 5′-GTC CAC CAC CCT GTT GCT GTA G-3 (reverse); α-SYN (rat), 5′-CCT CAG CCC AGA GCC TTT C-3′(forward), 5′-CCT CTG CCA CAC CCT GCT T-3′(reverse); GAPDH(mouse), 5′-GTT TCT TAC TCC TTG GAG GCC AT-3′(forward), 5′-TGA TGA CAT CAA GAA GTG GTG AA-3′(reverse); and α-SYN (mouse), 5′-GTG GTT CAT GGA GTG ACA AC-3′(forward), 5′-AGG CTT CAG GCT CAT AGT CT-3′ (reverse). PCR products were separated in 2% agarose gels and stained with Gelview. The band densities were analyzed with Image J software (National Institute of Health, USA).

### Statistical Analysis

Data are expressed as mean ± standard error from the mean (SEM). Differences between two groups were analyzed with Student’s *t*-test. Multiple comparisons among groups were performed using one-way analysis of variance followed by post-hoc analysis (Tukey’s). The statistical significance level was set at *P*<0.05.

## Results

### Systemic LPS Injection Induced Persistent Neuroinflammation and Delayed DA Neuron Loss and α-SYN Accumulation in the Midbrain

**Figure 1:**
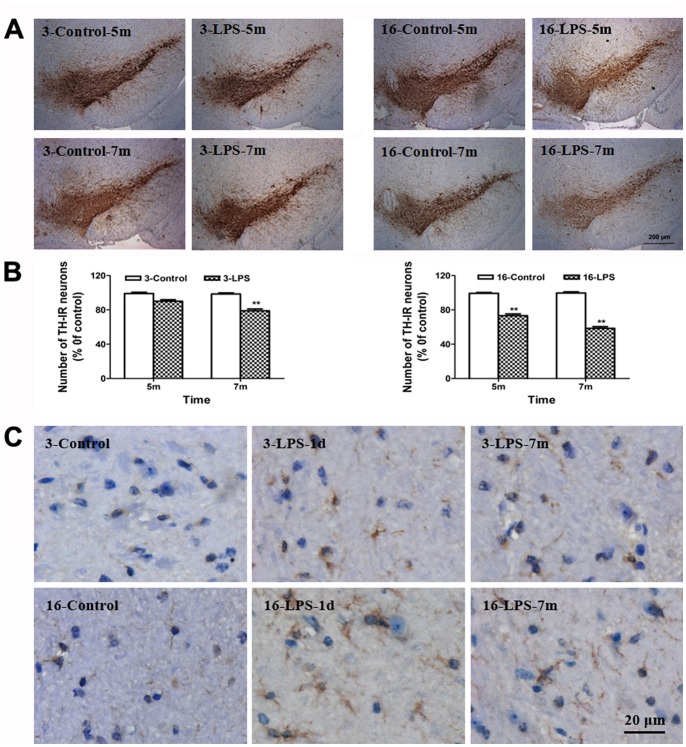
Systemic LPS injection caused progressive loss of DA neurons and persistent neuroinflammation in the midbrain. (A) The TH^+^ cells indicate the residual DA neurons. (B) The number of TH^+^ neurons in the SN of young and aged mice was counted at different time periods (5 m, and 7 m) after injection. (C) Activated microglia in the midbrain were visualized by increased cells size, irregular shape, and intensified Iba-1 staining. Results are represented as mean ± SEM, n  = 4. **P<0.01 compared to the corresponding saline-treated group at the same time after treatment (controls). Scale bar: 200 µm (A), 20 µm (C). 3-Control and 3-LPS represent young mice treated with saline and LPS; 16-Control and 16-LPS represent aged mice injected with saline and LPS, respectively. d, day; m, month. 1d, 5 m and 7 m indicate the time period after treatment.

The number of residual DA neurons and the activation of microglia were detected by immunohistochemistry. There was a delayed but significant loss of TH-positive neurons in LPS-treated young mice, which occurred at seven months after LPS injection (22% of TH-IR neuron loss), consistent with a previous report [11]. In aged mice, the loss of TH-IR neurons was more pronounced, approaching approximately 27% at five months, and further increased to 41% at seven months after LPS injection (Fig. 1A–B). In addition, as late as seven months after LPS treatment, Iba-1-positive cells in SN displayed increased cell size and irregular shape, consistent with morphological changes in response to microglial activation (Fig. 1C).

**Figure 2:**
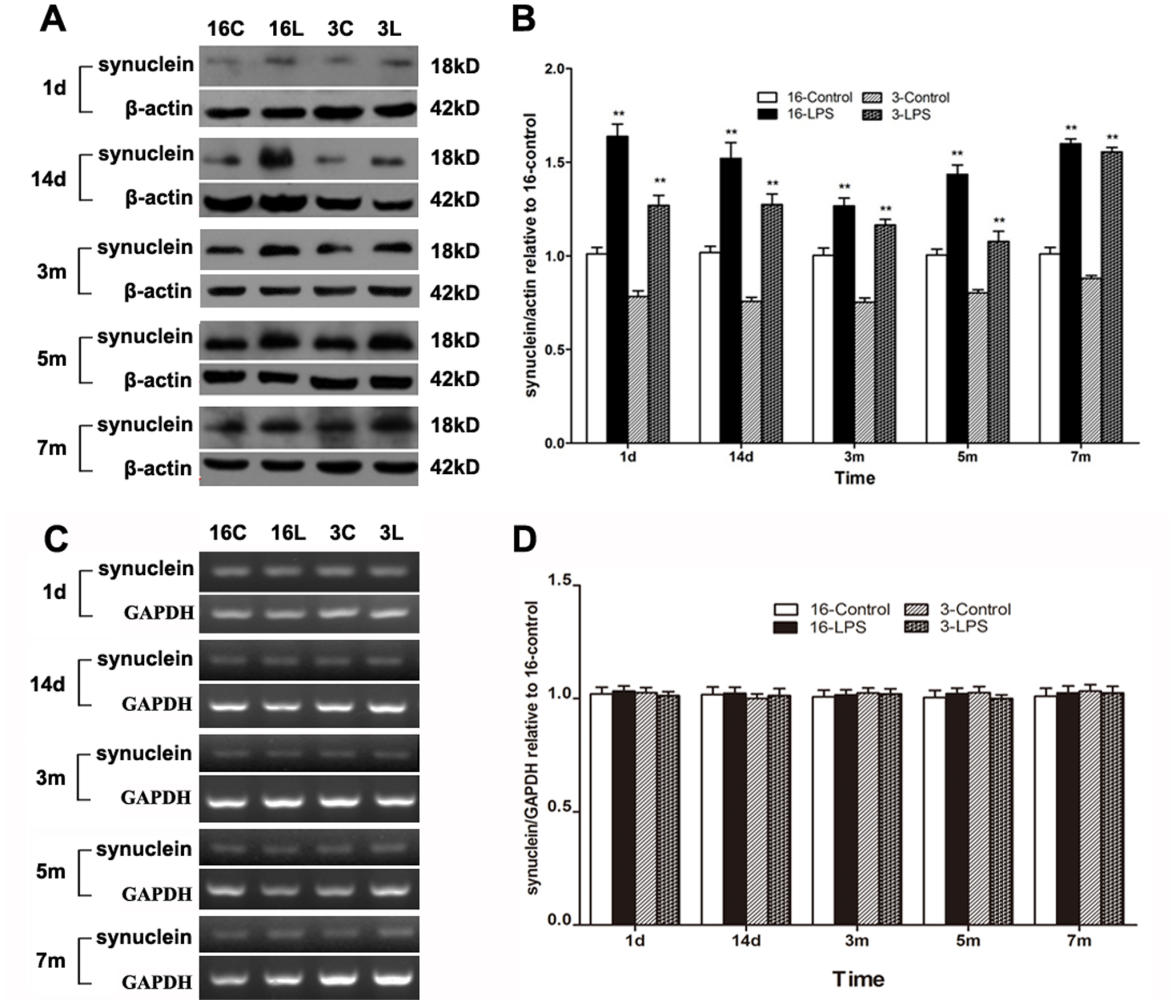
Systemic LPS injection induced α-SYN accumulation in the midbrain. The protein and mRNA levels of α-SYN were examined by Western blotting (A, B) and reverse transcription PCR (C, D) at different time periods (1 d, 14 d, 3 m, 5 m, and 7 m) after treatment. Representative gel results (A, C) and group data (B, D) indicated that compared with saline-treated mice, the α-SYN protein levels were increased in LPS-treated mice in both young and aged mice, whereas α-SYN mRNA levels remained unchanged. Results are represented as mean ± SEM, n  = 4. **P<0.01, compared to the corresponding saline-treated group at the same time after treatment (controls). d, day; m, month. 16C and 16L, aged mice injected with saline and LPS; 3C and 3L, young mice treated with saline and LPS, respectively.

α-SYN aggregation is closely related to DA neuron degeneration, and the formation of α-SYN-containing cytoplasmic inclusions is a hallmark of PD. Unfortunately, α-SYN-positive inclusions were absent in this study (data not shown). The α-SYN levels in the midbrain at different time periods after LPS injection were examined by Western blotting. α-SYN expression in LPS-treated mice was already higher than that in saline-treated animals as early as one day after treatment. This increase persisted until seven months after LPS injection (Fig. 2A–B). However, the α-SYN mRNA level was not altered in either young or aged mice (Fig. 2C–D).

### Systemic LPS Injection Caused Autophagic Impairment in the Midbrain

**Figure 3:**
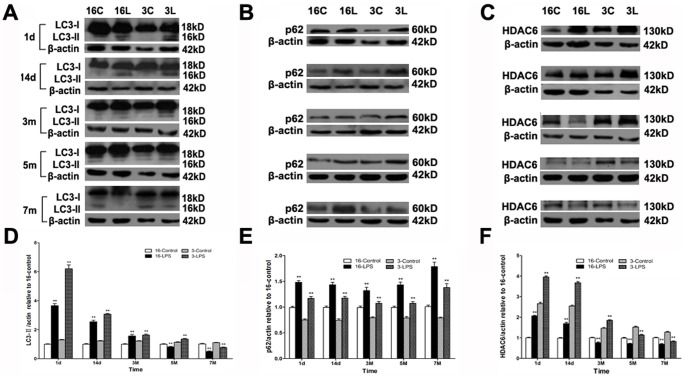
Systemic LPS injection caused autophagic impairment in the midbrain. The protein levels of LC3-II, p62, and HDAC6 were examined by Western blotting at different time points (1 d, 14 d, 3 m, 5 m, and 7 m) after LPS injection. (A-C) Representative blots demonstrate the levels of LC3-II, p62, and HDAC6. (D, F) Compared with saline-treated mice, there were time-dependent changes in LC3-II and HDAC6 expression in both young and aged LPS-treated mice. (E) Compared with saline-treated mice, p62 levels increased persistently in both young and aged LPS-treated mice. Results are represented as mean ± SEM, n  = 4. **P<0.01 compared to the corresponding saline-treated group at the same time after treatment (controls).

Autophagic activity was evaluated by examining the protein expression of autophagic markers, including microtubule-associated protein 1 light chain 3-II (LC3-II) and p62. Western blotting indicated differentially time-dependent changes in the LC3-II and p62 levels of young and aged mice after LPS injection. In young mice, LC3-II was higher from one day to five months after LPS injection but markedly reduced at seven months after injection compared to saline-treated mice. Similar trends were observed in aged mice. However, the decrease in aged mice occurred earlier, starting at five months and continuing until seven months after LPS injection (Figs. 3A & 3D). Interestingly, in both young and aged mice, the p62 level was persistently increased in the LPS-injected group compared to saline-treated mice (Figs. 3B & 3E).

HDAC6 recognizes damaged proteins and controls the fusion of autophagosomes to lysosomes where misfolded proteins are degraded [16]. Western blotting analysis demonstrated that in contrast to saline-treated mice, HDAC6 levels in the LPS-treated groups also exhibited distinct time-dependent changes in young and aged mice. The HDAC6 expression in LPS-injected young mice was significantly higher than in controls from one day to three months after injection, but it was reduced at five months and later. Compared with the young groups, the decrease of HDAC6 in aged mice occurred earlier, starting from three months and continuing up to seven months after treatment (Figs. 3C & 3F).

### Systemic LPS Injection Induced Autophagic Impairment in Midbrain DA Neurons

**Figure 4:**
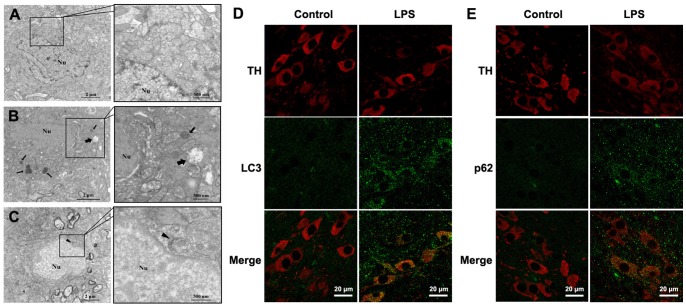
Systemic LPS injection induced autophagic impairment in midbrain DA neurons. Electron microscopic examination revealed ultrastructural changes in midbrain slices at one day after saline or LPS treatment in young mice. (A) Normal structure of a neuron in young mice receiving saline injection. (B) More lysosomes and autolysosomes were observed after LPS treatment. (C) Mitophagy, the specific autophagic elimination of mitochondria, was also detected in neurons. At one day after injection, LPS induced discernible increase in both LC3 (green, D) and p62 (green, E) in DA neurons. Pictures were observed and taken with a confocal microscope. Arrowheads: mitophagy; Thin arrows: lysosomes; Thick arrows: autolysosomes; Nu: Cell nuclei. Scale bar: 2 µm, 0.5 µm (A-C), 20 µm (D, E).

Visualization of autophagic vesicles is the gold standard of autophagy activation [20]. We sought to verify our findings by electron microscopy to confirm the existence of autophagic induction in midbrain at one day after LPS treatment in young mice. The neurons in saline-treated mice displayed nuclear membrane integrity, normal chromatin structure, and cytoplasmic organelles (Fig. 4A). More lysosomes and autolysosomes were observed after LPS treatment (Fig. 4B), indicating the induction of autophagy. In addition, mitophagy, the specific autophagic elimination of injured mitochondria, was also detected in the midbrain neurons (Fig. 4C).

To define the autophagic changes in DA neurons, we performed an immunohistochemistry study using specific markers. LC3 staining (green) was detected in TH-positive neurons (red) in the midbrain one day after LPS treatment in young mice (Fig. 4D). Furthermore, p62 staining (green) was observed in TH-positive neurons (red) (Fig. 4E). These results confirmed the autophagic impairment of DA neurons after LPS treatment.

### TNF-α Caused Autophagic Impairment and Cell Injury in PC12 Cells

**Figure 5:**
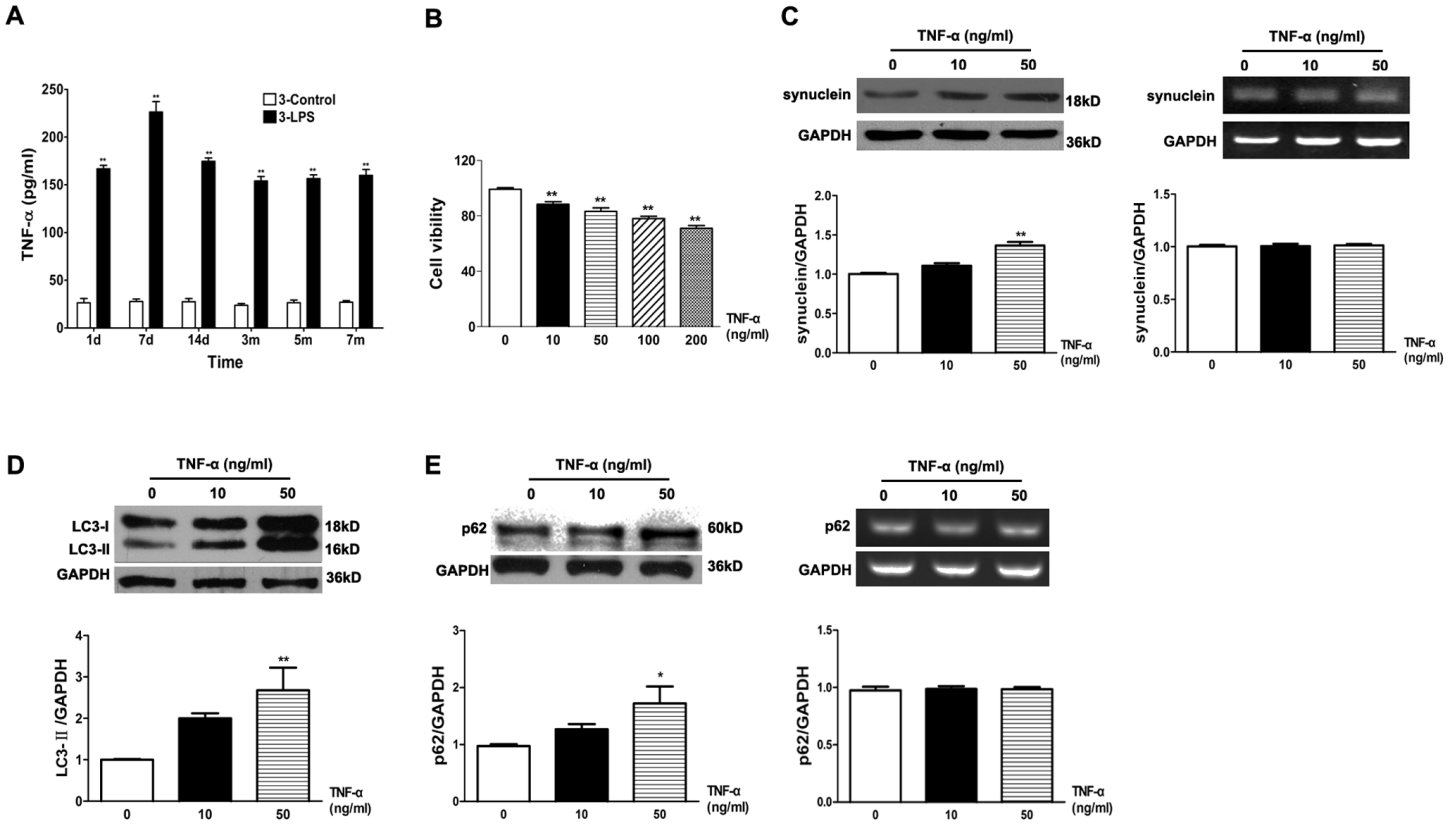
TNF-α reduced the cell survival and induced the autophagic dysfunction in PC12 cells. (A) TNF-α levels were persistently higher in young mouse midbrains after LPS injection. (B) Concentration-dependent effects of TNF-α (10, 50, 100, and 200 ng/ml) treatment for 24 h on the viability of PC12 cells, which was determined by the MTT method. The protein and mRNA levels of α-SYN (C) and p62 (E) were examined by Western blotting and reverse transcription PCR after treatment with TNF-α for 24 h in PC12 cells. (D) TNF-α (50 ng/ml) caused a marked increase of LC3-II as determined by Western blotting. Results are normalized to the value in control group (TNF 0 ng/ml) and shown as mean ± SEM, n  = 4–8. *P<0.05, **P<0.01 compared to controls.

To verify the involvement of autophagic regulation in LPS-treated mice, we further examined the effect of TNF-α on the autophagy pathway *in vitro* because TNF-α is an important pro-inflammatory cytokine released by activated glia. Nigral DA neurons are extremely sensitive to TNF-α stimulation [21]. Qin and his coworkers demonstrated that TNF-α caused DA neuron loss in mice intraperitoneally injected with LPS [11]. We also found that the midbrain TNF-α level in LPS-injected young mice was higher than that in saline-treated group over the entire period of observation (Fig. 5A). The viability of PC12 cells (a DA cell line) was determined with the MTT reduction assay. We found that after exposure to TNF-α treatment (10, 50, 100, and 200 ng/ml) for 24 h, the cell viability decreased significantly in a concentration-dependent manner (Fig. 5B). The sublethal dose of TNF-α (50 ng/ml) caused a significant accumulation of α-SYN protein in PC12 cells as revealed in Western blotting analyses. Reverse transcription PCR demonstrated that α-SYN mRNA levels remained unaltered after TNF-α treatment (Fig. 5C), indicating that the α-SYN increase might result from impaired clearance rather than transcriptional upregulation. Western blots further indicated that the sublethal dose of TNF-α (50 ng/ml) caused a significant upregulation of autophagosome marker LC3-II in PC12 cells (Fig. 5D). Furthermore, treatment of TNF-α (50 ng/ml) for 24 h increased the level of p62 protein. The mRNA level of p62 did not change after TNF-α treatment (Fig. 5E), although infection has been reported to induce its transcription in some studies [22]. These results suggested that TNF-α might trigger autophagic impairment associated with α-SYN protein accumulation in PC12 cells.

## Discussion

Macrophagy is a major evolutionarily conserved response to nutrient and bioenergetic depletion. Alterations in autophagic activities are implicated in a variety of disorders, including cancer, stroke, and neurodegenerative diseases [23]–[25]. Increasing evidence suggests ALP is affected in PD [26]. However, whether autophagy is beneficial or detrimental to disease progression depends largely on the specific situation and the stage of the pathological process [27]. Thus, to delay or prevent the onset or progression of PD, it is important to understand the events by which autophagic function is affected during PD progression. Inflammation is implicated as one of the contributors to DA neuron damage in PD [28], [29]. In this study, we found that systemic LPS injection induced persistent neuroinflammation and DA neuron loss in the midbrain. Although α-SYN inclusions were not found in our study (data not shown), we found a significant midbrain increase of α-SYN protein, but not mRNA, after LPS treatment. Moreover, the α-SYN increase was more evident later, at seven months after LPS injection, when a dramatic loss of DA neurons was observed in the midbrain. These data indicated that LPS treatment might cause an impairment of α-SYN clearance, leading to its pathological accumulation, particularly in the later phase.

LC3, which is converted from LC3-I to LC3-II via covalent conjugation with phosphatidylethanolamine and recruited to phagophores or isolated membranes, is frequently used as an autophagosome marker [30]. p62 is one of the specific substrates that are degraded via autophagy because it has a ubiquitin-associated domain and an LC3-interacting region [31]. Ablation of autophagy leads to p62 accumulation [32]. Similar p62-positive cytoplasmic inclusions were found in several neurodegenerative diseases, including PD [33]. HDAC6 controls the fusion of autophagosomes to lysosomes. Deficiencies in HDAC6 result in the accumulation of protein aggregates and in neurodegeneration [16]. Thus, HDAC6 is a key player in the pathogenesis of neurodegenerative disorders. We found that the levels of LC3-II and HDAC6 expression changed dynamically but that p62 increased persistently in the midbrain during the entire process after LPS injection. In both young and aged mice, LC3-II and HDAC6 levels were increased in the early period (one day and 14 days after LPS treatment) followed by a decline at late stages. Moreover, p62 was observed to remain at a higher level compared to the controls, and it continued to increase, whereas LC3-II and HDAC6 protein levels were reduced at five and seven months after treatment. These data indicated an impairment in autophagic flux as inflammation progressed and persisted. HDAC6 activity is essential for autophagy to compensate for impaired UPS function [17]. Our observations that HDAC6 expression increased synchronously with LC3-II in the early period and that it decreased prior to LC3-II at late stage supported a critical role for HDCA6 in the autophagic response.

The activation of autophagy may be a protective mechanism against inflammatory insults; however, disruption of autophagic flux would induce the accumulation of misfolded proteins, such as α-SYN, that eventually results in neuronal death. As inflammation persists, long periods of autophagic activation may decrease autophagosome formation due to phagophore depletion or other factors. This trend is reflected by early increases in LC3-II and HDAC6, followed by decreases in their expression. Neuronal autophagy is essential for protein homeostasis, and autophagy dysfunction increases the accumulation of potentially pathogenic α-SYN oligomers [34]. Autophagy-deficient DA neurons in aged animals were susceptible to presynaptic α-SYN accumulation. This susceptibility may explain our observations that the α-SYN increase was more discernible in the later stage, when LC3-II and HDAC6 were already decreased. These results suggest that disrupted autophagy may be associated with increased levels of endogenous α-SYN proteins *in vivo*. Thus, we propose that inflammation may trigger the activation of autophagy but that persistent inflammation may lead to autophagic impairment in the midbrain, which in turn results in the accumulation of endogenous α-SYN. In fact, α-SYN could further trigger neuroinflammatory responses by activating microglia [35]. Therefore, pathological α-SYN accumulation and neuroinflammation may synergistically contribute to DA neuron degeneration.

Neurons typically maintain autophagic activity at a basal level to remove damaged organelles and misfolded proteins. A variety of stress-related signals cause rapid formation and accumulation of autophagosomes in neurons [36]. Electron microscopic examinations here demonstrated the appearance of autophagosomes in midbrain neurons at one day after LPS treatment. The immunostaining results further revealed the upregulation of LC3 and p62 in the residual DA neurons, indicating that systemic LPS exposure impaired autophagic flux in DA neurons. As activated microglia may release many inflammatory cytokines in response to LPS treatment, certain pro-inflammatory cytokine(s) may be involved in the autophagic impairment mechanisms.

TNF-α is the main pro-inflammatory cytokine released by glial cells and has been shown to serve as a critical mediator of nigral DA neuron death during neurodegeneration. We also found that the TNF-α level was elevated in the midbrain after LPS injection. The *in vitro* study further demonstrated that TNF-α caused neurotoxicity to DA cells. Moreover, TNF-α markedly increased the protein levels of α-SYN without affecting α-SYN mRNA, indicating an impairment in clearance of this protein after TNF-α treatment. Meanwhile, the expression levels of LC3-II and p62 were increased in PC12 cells. A transcriptional increase may also contribute to the rise in p62 expression because LPS has been reported to be a strong inducer for its transcription [22]. However, our results for p62 mRNA levels excluded this possibility. As p62 is a substrate of ALP, its increase observed here indicates a disturbance in autophagic flux. These data indicate that TNF-α triggered autophagic impairment in PC12 cells and induced the accumulation of endogenous α-SYN. Thus, we propose that TNF-α acts not only as an inflammation mediator but also as a trigger of autophagic impairment and neuronal damage. However, the contribution of other inflammatory mediators cannot be completely excluded. It is likely that TNF-α, either alone or together with other factors, is sufficient to trigger autophagic dysfunction in DA neurons. This topic warrants further study.

The capacity for autophagy-mediated protein degradation declines with age [37]. The expression of several autophagy genes also decreases with age [38], [39]. In our study, the delayed loss of DA neurons appeared earlier in aged mice compared with young group. Similarly, the increase in α-SYN and p62, as well as the reduction in LC3-II and HDAC6, occurred earlier in aged mice. It indicated that systemic inflammation induces an age-influenced change in autophagic activity, α-SYN accumulation, and DA neuron loss in the midbrain. These results are consistent with previous reports that age-dependent suppression of autophagy is closely associated with the build-up of cellular damage in neurons [38].

Several procedures have been developed for LPS administration including intrastriatal, intrapallidal, intranigral and intraperitoneal injection. Local orientation injection may cause rapid but not progressive DA neuron loss in the nigrastriatal pathway. Compared with this, systemic LPS injection does not specifically affect the nigrastriatal pathway, but triggers neuroinflammation in several microglia densely populated brain regions including hippocampus and olfactory telencephalon [40]. Nevertheless, the oxidative damage-vulnerable DA neuron in SN is more susceptible to the neuroinflammation-mediated neurotoxicity. Thus, systemic (intraperitoneal) administration induces delayed and progressive DA neuron degeneration, which occurs at several months after LPS treatment, as we and other researchers demonstrated [11].

This study demonstrated the changes in autophagic activity and α-SYN accumulation during the entire process of a delayed and progressive loss of DA neurons in the midbrain caused by systemic inflammation. Our observations revealed that autophagic impairment contributes to the neurotoxicity on DA neurons induced by systemic inflammation. Thus, pharmacological regulation of autophagy may be a useful approach for PD therapy, but its utility depends on the disease progression.

## References

[1] Nguyen MD, Julien JP, Rivest S (2002) Innate immunity: the missing link in neuroprotection and neurodegeneration? Nat Rev Neurosci 3: 216–227. 11994753 10.1038/nrn752

[2] McGeer PL, Itagaki S, Boyes BE, McGeer EG (1988) Reactive microglia are positive for HLA-DR in the substantia nigra of Parkinson’s and Alzheimer’s disease brains. Neurology 38: 1285–1291. 3399080 10.1212/wnl.38.8.1285

[3] Czlonkowska A, Kohutnicka M, Kurkowska-Jastrzebska I, Czlonkowski A (1996) Microglial reaction in MPTP (1-methyl-4-phenyl-1,2,3,6-tetrahydropyridine) induced Parkinson’s disease mice model. Neurodegeneration 5: 137–143. 8819134 10.1006/neur.1996.0020

[4] Gao HM, Jiang J, Wilson B, Zhang W, Hong JS, et al (2002) Microglial activation-mediated delayed and progressive degeneration of rat nigral dopaminergic neurons: relevance to Parkinson’s disease. J Neurochem 81: 1285–1297. 12068076 10.1046/j.1471-4159.2002.00928.x

[5] Tomas-Camardiel M, Rite I, Herrera AJ, de Pablos RM, Cano J, et al (2004) Minocycline reduces the lipopolysaccharide-induced inflammatory reaction, peroxynitrite-mediated nitration of proteins, disruption of the blood-brain barrier, and damage in the nigral dopaminergic system. Neurobiol Dis 16: 190–201. 15207276 10.1016/j.nbd.2004.01.010

[6] Inamdar AA, Chaudhuri A, O’Donnell J (2012) The Protective Effect of Minocycline in a Paraquat-Induced Parkinson’s Disease Model in Drosophila is Modified in Altered Genetic Backgrounds. Parkinsons Dis 2012: 938528. 22900232 10.1155/2012/938528 PMC3413958

[7] Hakansson A, Westberg L, Nilsson S, Buervenich S, Carmine A, et al (2005) Investigation of genes coding for inflammatory components in Parkinson’s disease. Mov Disord 20: 569–573. 15648059 10.1002/mds.20378

[8] Infante J, Garcia-Gorostiaga I, Sanchez-Juan P, Sanchez-Quintana C, Gurpegui JL, et al (2008) Inflammation-related genes and the risk of Parkinson’s disease: a multilocus approach. Eur J Neurol 15: 431–433. 18284424 10.1111/j.1468-1331.2008.02092.x

[9] Xu X, Li D, He Q, Gao J, Chen B, et al (2011) Interleukin-18 promoter polymorphisms and risk of Parkinson’s disease in a Han Chinese population. Brain Res 1381: 90–94. 21241672 10.1016/j.brainres.2011.01.025

[10] Kim WG, Mohney RP, Wilson B, Jeohn GH, Liu B, et al (2000) Regional difference in susceptibility to lipopolysaccharide-induced neurotoxicity in the rat brain: role of microglia. J Neurosci 20: 6309–6316. 10934283 10.1523/JNEUROSCI.20-16-06309.2000 PMC6772569

[11] Qin L, Wu X, Block ML, Liu Y, Breese GR, et al (2007) Systemic LPS causes chronic neuroinflammation and progressive neurodegeneration. Glia 55: 453–462. 17203472 10.1002/glia.20467 PMC2871685

[12] Hoshino K, Takeuchi O, Kawai T, Sanjo H, Ogawa T, et al (1999) Cutting edge: Toll-like receptor 4 (TLR4)-deficient mice are hyporesponsive to lipopolysaccharide: evidence for TLR4 as the Lps gene product. J Immunol 162: 3749–3752. 10201887

[13] Lehnardt S, Massillon L, Follett P, Jensen FE, Ratan R, et al (2003) Activation of innate immunity in the CNS triggers neurodegeneration through a Toll-like receptor 4-dependent pathway. Proc Natl Acad Sci U S A 100: 8514–8519. 12824464 10.1073/pnas.1432609100 PMC166260

[14] Liu B, Gao HM, Wang JY, Jeohn GH, Cooper CL, et al (2002) Role of nitric oxide in inflammation-mediated neurodegeneration. Ann N Y Acad Sci 962: 318–331. 12076984 10.1111/j.1749-6632.2002.tb04077.x

[15] Lees AJ, Hardy J, Revesz T (2009) Parkinson’s disease. Lancet 373: 2055–2066. 19524782 10.1016/S0140-6736(09)60492-X

[16] Lee JY, Koga H, Kawaguchi Y, Tang W, Wong E, et al (2010) HDAC6 controls autophagosome maturation essential for ubiquitin-selective quality-control autophagy. EMBO J 29: 969–980. 20075865 10.1038/emboj.2009.405 PMC2837169

[17] Pandey UB, Nie Z, Batlevi Y, McCray BA, Ritson GP, et al (2007) HDAC6 rescues neurodegeneration and provides an essential link between autophagy and the UPS. Nature 447: 859–863. 17568747 10.1038/nature05853

[18] Hara T, Nakamura K, Matsui M, Yamamoto A, Nakahara Y, et al (2006) Suppression of basal autophagy in neural cells causes neurodegenerative disease in mice. Nature 441: 885–889. 16625204 10.1038/nature04724

[19] Komatsu M, Waguri S, Chiba T, Murata S, Iwata J, et al (2006) Loss of autophagy in the central nervous system causes neurodegeneration in mice. Nature 441: 880–884. 16625205 10.1038/nature04723

[20] Mizushima N (2004) Methods for monitoring autophagy. Int J Biochem Cell Biol 36: 2491–2502. 15325587 10.1016/j.biocel.2004.02.005

[21] McGuire SO, Ling ZD, Lipton JW, Sortwell CE, Collier TJ, et al (2001) Tumor necrosis factor alpha is toxic to embryonic mesencephalic dopamine neurons. Exp Neurol 169: 219–230. 11358437 10.1006/exnr.2001.7688

[22] Fujita K, Maeda D, Xiao Q, Srinivasula SM (2011) Nrf2-mediated induction of p62 controls Toll-like receptor-4-driven aggresome-like induced structure formation and autophagic degradation. Proc Natl Acad Sci U S A 108: 1427–1432. 21220332 10.1073/pnas.1014156108 PMC3029726

[23] Carew JS, Kelly KR, Nawrocki ST (2012) Autophagy as a target for cancer therapy: new developments. Cancer Manag Res 4: 357–365. 23091399 10.2147/CMAR.S26133 PMC3474143

[24] Gabryel B, Kost A, Kasprowska D (2012) Neuronal autophagy in cerebral ischemia–a potential target for neuroprotective strategies? Pharmacol Rep 64: 1–15. 22580515 10.1016/s1734-1140(12)70725-9

[25] Cheung ZH, Ip NY (2011) Autophagy deregulation in neurodegenerative diseases - recent advances and future perspectives. J Neurochem 118: 317–325. 21599666 10.1111/j.1471-4159.2011.07314.x

[26] Lynch-Day MA, Mao K, Wang K, Zhao M, Klionsky DJ (2012) The role of autophagy in Parkinson’s disease. Cold Spring Harb Perspect Med 2: a009357. 22474616 10.1101/cshperspect.a009357 PMC3312403

[27] Rubinsztein DC, DiFiglia M, Heintz N, Nixon RA, Qin ZH, et al (2005) Autophagy and its possible roles in nervous system diseases, damage and repair. Autophagy 1: 11–22. 16874045 10.4161/auto.1.1.1513

[28] Kim YS, Joh TH (2006) Microglia, major player in the brain inflammation: their roles in the pathogenesis of Parkinson’s disease. Exp Mol Med 38: 333–347. 16953112 10.1038/emm.2006.40

[29] Block ML, Zecca L, Hong JS (2007) Microglia-mediated neurotoxicity: uncovering the molecular mechanisms. Nat Rev Neurosci 8: 57–69. 17180163 10.1038/nrn2038

[30] Kabeya Y, Mizushima N, Ueno T, Yamamoto A, Kirisako T, et al (2000) LC3, a mammalian homologue of yeast Apg8p, is localized in autophagosome membranes after processing. EMBO J 19: 5720–5728. 11060023 10.1093/emboj/19.21.5720 PMC305793

[31] Komatsu M, Ichimura Y (2010) Physiological significance of selective degradation of p62 by autophagy. FEBS Lett 584: 1374–1378. 20153326 10.1016/j.febslet.2010.02.017

[32] Komatsu M, Waguri S, Koike M, Sou YS, Ueno T, et al (2007) Homeostatic levels of p62 control cytoplasmic inclusion body formation in autophagy-deficient mice. Cell 131: 1149–1163. 18083104 10.1016/j.cell.2007.10.035

[33] Kuusisto E, Salminen A, Alafuzoff I (2001) Ubiquitin-binding protein p62 is present in neuronal and glial inclusions in human tauopathies and synucleinopathies. Neuroreport 12: 2085–2090. 11447312 10.1097/00001756-200107200-00009

[34] Yu WH, Dorado B, Figueroa HY, Wang L, Planel E, et al (2009) Metabolic activity determines efficacy of macroautophagic clearance of pathological oligomeric alpha-synuclein. Am J Pathol 175: 736–747. 19628769 10.2353/ajpath.2009.080928 PMC2716969

[35] Lee EJ, Woo MS, Moon PG, Baek MC, Choi IY, et al (2010) Alpha-synuclein activates microglia by inducing the expressions of matrix metalloproteinases and the subsequent activation of protease-activated receptor-1. J Immunol 185: 615–623. 20511551 10.4049/jimmunol.0903480

[36] Chu CT (2006) Autophagic stress in neuronal injury and disease. J Neuropathol Exp Neurol 65: 423–432. 16772866 10.1097/01.jnen.0000229233.75253.be PMC1885377

[37] Vittorini S, Paradiso C, Donati A, Cavallini G, Masini M, et al (1999) The age-related accumulation of protein carbonyl in rat liver correlates with the age-related decline in liver proteolytic activities. J Gerontol A Biol Sci Med Sci 54: B318–323. 10496537 10.1093/gerona/54.8.b318

[38] Simonsen A, Cumming RC, Brech A, Isakson P, Schubert DR, et al (2008) Promoting basal levels of autophagy in the nervous system enhances longevity and oxidant resistance in adult Drosophila. Autophagy 4: 176–184. 18059160 10.4161/auto.5269

[39] Shibata M, Lu T, Furuya T, Degterev A, Mizushima N, et al (2006) Regulation of intracellular accumulation of mutant Huntingtin by Beclin 1. J Biol Chem 281: 14474–14485. 16522639 10.1074/jbc.M600364200

[40] Lawson LJ, Perry VH, Dri P, Gordon S (1990) Heterogeneity in the distribution and morphology of microglia in the normal adult mouse brain. Neuroscience 39: 151–170. 2089275 10.1016/0306-4522(90)90229-w

